# Burnout and turnover intention in primary public health workers: the mediating role of job satisfaction

**DOI:** 10.3389/fpubh.2025.1708432

**Published:** 2026-01-15

**Authors:** Zongliang Wen, Yiheng Qin, Yuting Ni, Yan Wang, Keyue Xu, Yan Zhu

**Affiliations:** 1 School of Management, Xuzhou Medical University, Xuzhou, China; 2 Institution of Chinese Health Modernization, Xuzhou, China; 3 Affiliated Hospital of Xuzhou Medical University, Xuzhou, China; 4 School of Medical Information and Engineering, Xuzhou Medical University, Xuzhou, China

**Keywords:** burnout, job satisfaction, turnover intention, primary public health workers, mediating effect

## Abstract

**Background:**

With increasing attention to primary health care across countries worldwide, primary public health workers are being assigned expanding responsibilities, resulting in heightened workloads and psychological strain. This has contributed to widespread burnout and heightened turnover risk.

**Objective:**

Guided by the Job Demands-Resources (JD-R) model, this study aimed to observe turnover intention among primary public health workers and explore the interrelationships between burnout, job satisfaction, and turnover intention.

**Methods:**

A cross-sectional survey was conducted among 6,725 primary public health workers in the Huaihai Economic Zone in China to assess their levels of burnout, job satisfaction, and turnover intention. One-way ANOVA was used to examine group differences, stepwise multiple linear regression identified influencing factors, and structural equation modeling (AMOS) test the mediating role of job satisfaction.

**Results:**

Burnout was negatively associated with job satisfaction (*r* = −0.557) and positively with turnover intention (*r* = 0.475, both *p* < 0.001), while job satisfaction was inversely related to turnover intention (*r* = −0.429, *p* < 0.001). Regression analysis identified burnout (*β* = 0.337, *p* < 0.001) and job satisfaction (*β* = −0.239, *p* < 0.001) as key predictors of turnover intention. Structural equation modeling further indicated that job satisfaction partially mediated the link between burnout and turnover intention (indirect effect = 0.089, 95% CI: 0.066–0.112), explaining 22% of the total effect.

**Conclusion:**

The study revealed that burnout influences turnover intention both directly and indirectly, with job satisfaction serving as a key psychological pathway. Addressing burnout requires multi-level strategies—enhancing individual motivation, strengthening organizational support, and advancing institutional reforms—to mitigate psychological strain and sustain the stability of the primary public health workforce.

## 1 Introduction

Primary public health workers are a cornerstone of global health systems, essential for achieving universal health coverage, promoting health equity, and responding effectively to public health emergencies. As the “first line of defense” in healthcare, they provide core services such as disease prevention, health education, and chronic disease management, while also playing critical roles in resident health oversight, family doctor contracting, and referral coordination. These workers are not only direct service providers but also key facilitators of equitable healthcare access. In China, they are pivotal to the “Healthy China” strategy, forming the core of the public health delivery system through work in community and township health centers, where they offer essential services including maternal and child care, chronic disease control, and population-level health promotion (1). While not directly equivalent, similar roles internationally include community health workers (CHWs), public health officers (PHOs), and primary care providers with public health responsibilities (2–4). As China promotes its “primary-level health services” policy, these workers face expanding responsibilities and increasing occupational stress (5). This issue is not unique to China. Global studies consistently report high burnout risks among frontline public health workers due to heavy workloads, insufficient resources, and unclear role expectations, all of which compromise service quality and workforce retention (6, 7). In the Chinese context, empirical research has reported burnout rates among health professionals ranging from 66.5% to 76.9% (8–10). The psychological and workload burdens borne by primary public health workers are particularly severe, rendering occupational burnout a pressing issue that warrants urgent attention.

Burnout is a psychological response to prolonged work-related stress, characterized by emotional, cognitive, and behavioral exhaustion. In primary public health workers, it reduces job satisfaction, performance, and well-being, and is a key predictor of turnover intention (11). Conceptually, burnout encompasses three dimensions: emotional exhaustion (EE), depersonalization (DP), and reduced personal accomplishment (PA). EE reflects fatigue from sustained emotional demands; DP denotes emotional distancing from service recipients; and PA indicates a perceived decline in competence and professional efficacy (12). Numerous international studies have demonstrated that burnout and its three dimensions significantly predict turnover intention (13–15). For instance, Xu et al. (16) found that all three dimensions significantly and positively predicted turnover intention among 1,326 primary healthcare workers in Shanghai during the COVID-19 pandemic.

Turnover intention refers to an employee’s conscious and deliberate willingness to leave their current job, formed through a comprehensive evaluation of job satisfaction, interpersonal relationships, compensation, and career prospects. Research has found that the turnover intention of primary public health workers is influenced by multiple factors, including professional identity, work environment, burnout, job satisfaction, social support, and stress. Studies across various countries show a significant positive correlation between burnout and turnover intention; that is, the higher the level of burnout among healthcare workers, the stronger their intention to leave (17, 18). According to data from China’s National Health Commission, the turnover rate of physicians in public hospitals nationwide was 4.3% in 2022, with 67% of those leaving being under 35 years old. A meta-analysis involving 37,672 primary healthcare workers found that 30.4% expressed a willingness to leave (19). Since 2016, 66 countries have reported community health worker data to the WHO’s Workforce Account platform, with the latest total standing at only 2.4 million (20). The shortage of primary public health workers has become an increasingly severe issue in global health systems. The WHO projects a global shortfall of approximately 18 million primary health workers by 2030, particularly acute in low-income and developing regions. Therefore, timely and accurate identification of turnover intentions, along with proactive measures to maintain workforce stability in primary healthcare systems, is critically important.

However, existing studies have mostly focused on the direct relationship between burnout and turnover intention, with less attention to the underlying mechanisms. To provide a comprehensive occupational health perspective, this study draws on the Job Demands-Resources (JD-R) model. Originally proposed by Demerouti et al. (21) and later refined by Bakker and Demerouti (22), the JD-R model explains how the balance between job demands and available resources shapes employee well-being. Each occupation presents a unique combination of demands and resources. High demands, such as workload and administrative responsibilities, can deplete energy and contribute to burnout, whereas sufficient resources, including autonomy, social support, and recognition, foster motivation, engagement, and job satisfaction. Job satisfaction refers to an individual’s emotional evaluation of their work experience (23). Based on the JD-R model, we posit that job satisfaction functions as a resource-based pathway mediating the effect of burnout on turnover intention. Applied to China’s primary public health workforce, which faces chronic staff shortages and multiple role demands, this framing helps explain how structural resource constraints may amplify burnout’s direct effect on turnover intention while limiting satisfaction’s protective role. To further explain the psychological mechanism underlying this mediation, we draw on Self-Determination Theory (SDT) (24) and Social Exchange Theory (SET) (25). According to SDT, job satisfaction depends on the fulfillment of autonomy, competence, and relatedness needs. Burnout, especially emotional exhaustion and depersonalization, weakens these needs and lowers intrinsic motivation (26). Meanwhile, SET further suggests that support, recognition, and fair treatment from organizations promote trust, commitment, and satisfaction (25). Thus, job satisfaction connects psychological states with external resources and mediates the effect of burnout on turnover intention. This role has been confirmed across various occupational groups (27–29). For example, Yulia et al. (30) found job satisfaction and burnout fully mediated the association between professional identity and turnover intention among Israeli nurses post-pandemic. Integrating the JD-R model with SDT and SET, this study conceptualizes job satisfaction as the mechanism linking burnout and turnover intention through both resource-based and psychological-need pathways.

In recent years, studies have increasingly adopted a more comprehensive approach to examining the interrelationships among burnout, job satisfaction, turnover intention, and various mediating factors (31). However, notable limitations persist regarding sample selection. Much of the existing evidence has focused on healthcare professionals working in tertiary hospitals, with insufficient attention to the experiences of primary public health personnel. Unlike hospital-based clinicians and nursing staff, primary public health workers combine preventive and population-health duties with routine clinical tasks, often shoulder heavy administrative and reporting burdens, work under more limited material and human resources, and face less transparent promotion pathways and weaker financial incentive. As global health challenges grow, primary healthcare workers face heavier workloads and limited resources. Research should increasingly focus on this workforce, especially in low- and middle-income settings, to inform policies for workforce stability and health equity. In China, most studies draw samples from economically developed eastern provinces, with few adopting a cross-regional perspective. This limits understanding of systemic challenges and reduces the generalizability of findings. To address this gap, the present study focuses on the Huaihai Economic Zone as the sampling area. Spanning four provinces—Jiangsu, Shandong, Henan, and Anhui—this region is densely populated yet economically underdeveloped, with a relatively weak public health infrastructure. Cities within the zone exhibit notable disparities in both economic development and healthcare resource distribution. The region shows clear variation in socioeconomic development, healthcare resources, and the degree of urban–rural integration. It represents a microcosm of China’s grassroots public health system and provides an appropriate setting for studying workforce challenges in transitional contexts. Cities within the zone display notable disparities in both economic development and healthcare service capacity: some are relatively advanced, while others remain underdeveloped or underserved. Meanwhile, urban–rural intermixing is particularly evident in areas where expanding urban fringes border rural communities. Collectively, these features reflect a complex landscape of regional diversity and governance heterogeneity, making the findings not only representative within China but also informative for other regions worldwide facing uneven development and constrained health resources. Notably, recent evidence highlights pronounced strain among primary public health personnel, manifested as high burnout, low job satisfaction, and elevated turnover intention (32). Although these findings provide critical descriptive insights, they offer limited understanding of the mechanisms linking burnout to workforce attrition. Extending this work, the present study explicitly models job satisfaction as a mediating mechanism between burnout and turnover intention. This approach clarifies the psychological processes sustaining workforce stability and, by integrating a cross-regional governance perspective within a mechanism-based analytical framework, delivers both theoretical advancement and practical guidance for policy and workforce management.

**Figure 1:**
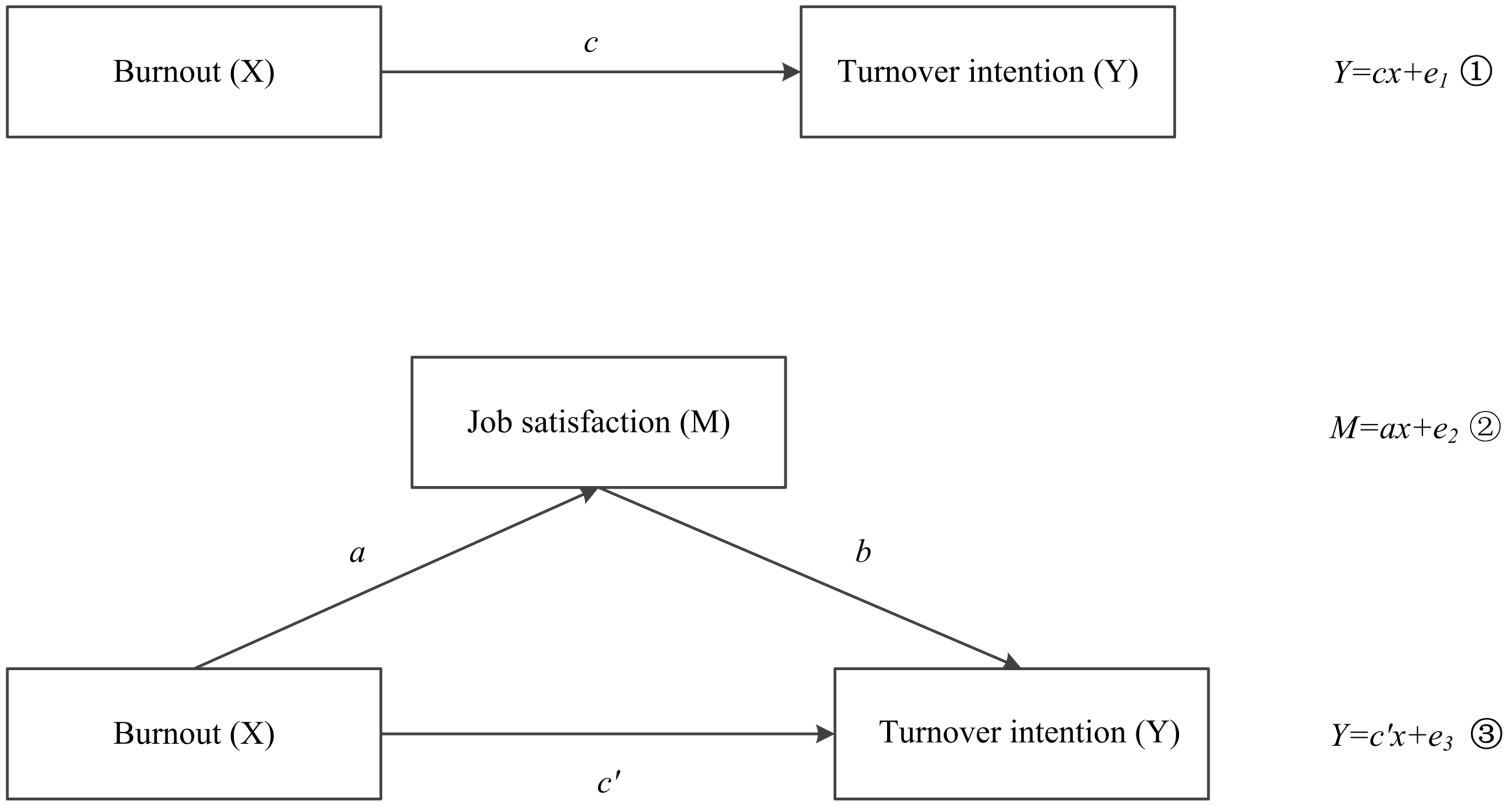
The conceptual model.

Overall, this study aims to examine the current status of burnout, job satisfaction, and turnover intention among primary public health workers, explore the relationships among these variables, and investigate the mediating role of job satisfaction to better identify underlying mechanisms. Based on the findings, we propose a multi-level intervention framework across individual, organizational, and societal dimensions to inform targeted strategies. Drawing on the literature, we formulated the following hypotheses (Figure 1):

*H1*: Burnout may be associated with turnover intention.

*H2*: Job satisfaction may affect turnover intention.

*H3*: Job satisfaction may mediate the relationship between burnout and turnover intention.

## 2 Materials and method

### 2.1 Participants and procedures

The study was conducted from February to March 2023 in China’s Huaihai Economic Zone, a region spanning the junction of Jiangsu, Anhui, Henan, and Shandong provinces. Characterized by a dense population, significant regional disparities, and a weak public health infrastructure amid ongoing urban–rural integration, the area offers a representative context for understanding structural challenges in China’s primary public health system and has broader relevance for policy translation. To represent diverse local conditions within the region, this study selected four cities—Xuzhou, Huaibei, Shangqiu, and Linyi: Xuzhou, as the regional center, features abundant medical resources and a well-established primary care system, serving as a benchmark; Huaibei reflects the urban–rural divide and diverse service needs Shangqiu represents rural healthcare status and integration of traditional Chinese medicine; and Linyi demonstrates the outcomes of healthcare coverage under health poverty alleviation initiatives.

A two-stage research design with stratified cluster sampling was employed. In the first stage, four primary healthcare institutions in Xuzhou were selected for in-depth field interviews and on-site observations, which informed the refinement of the questionnaire. In the second stage, an online survey was distributed, beginning with primary public health workers across urban and rural districts in Xuzhou, and later expanding to 194 township hospitals (including community hospitals) in Linyi; 16 community health centers in Huaibei; and 216 basic public health institutions in Shangqiu to comprehensively capture regional data. The sample spans urban, peri-urban and rural settings within the Huaihai Economic Zone and captures diverse institutional contexts, supporting the study’s regional representativeness. The sample spans urban, peri-urban and rural settings within the Huaihai Economic Zone and captures diverse institutional contexts, supporting the study’s regional representativeness.

The questionnaire, administered via the Wenjuanxing platform, was distributed through QR codes or survey links sent to participants’ mobile devices. It included the Turnover Intention Scale, Maslach Burnout Inventory–General Survey (MBI-GS), Minnesota Job Satisfaction Questionnaire (MQS), and demographic items. All scales were administered in Chinese and underwent a standard translation and back-translation procedure to ensure linguistic accuracy and cultural equivalence. Informed consent was obtained online. Responses with missing data or abnormal answering patterns were excluded. A total of 7,393 questionnaires were distributed, yielding 6,725 valid responses, resulting in a valid response rate of 90.96%.

Participants were included if they provided informed consent and had no known mental or cognitive impairments. Exclusion criteria included being on long-term leave (≥6 months) or currently engaged in internship or study-related activities. The study received ethical approval from the Ethics Committee of Xuzhou Medical University (ID: 225281) and was conducted in accordance with the principles of the Declaration of Helsinki (1989 revision).

### 2.2 Baseline characteristics of primary public health workers

The baseline demographics of frontline public health workers included gender (male, female), age groups (≤25, 26–30, 31–35, 36–40, 41–45, 46–50, 51–55, 56–60, ≥61), education (technical school, associate, bachelor master and doctor), technical title (senior, vice-senior, intermediate, junior, primary, no title), monthly income [≤3,000 yuan (≤US $430.9), 3,001–5,000 yuan (US $430.9–$718.2), 5,001–8,000 yuan (US $718.2–$1,104.8), ≥8,001 yuan (≥US $1,104.8)], employment status (formal staff, non-formal), years of work (≤2, 3–5, 6–9, ≥10) and daily working hours (≤8, 8–12, >12 h).

### 2.3 Burnout

The revised Maslach Burnout Inventory–General Survey (MBI-GS) was used to assess burnout among primary public health workers (33). The Chinese version of the MBI-GS has been validated and widely applied in healthcare populations, demonstrating good psychometric properties (34). Developed within the framework of Maslach’s burnout theory, this internationally recognized instrument is widely applied in healthcare settings. The scale includes three dimensions: Emotional Exhaustion (9 items), Depersonalization (5 items), and Perceived Achievement (8 items), totaling 22 items. Responses are rated on a 7-point Likert scale from 0 (“never”) to 6 (“daily”). In this study, the scale demonstrated good reliability, with a Cronbach’s *α* coefficient of 0.878 and a KMO value of 0.932, indicating its high validity.

### 2.4 Job satisfaction

Job satisfaction among primary public health workers was assessed using the revised Minnesota satisfaction scale, which measures two aspects of job satisfaction: internal satisfaction, which evaluates employees’ satisfaction with intrinsic job factors, and external satisfaction, which assesses satisfaction with external factors such as pay, rewards, promotion opportunities, organizational policies, and leadership style. It uses a 5-point Likert scale ranging from 1 to 5, where scores closer to 1 indicate lower satisfaction and scores closer to 5 indicate higher satisfaction (35). The Minnesota satisfaction scale has been widely used in healthcare research, and a validated Chinese version has been applied among primary healthcare workers (36). Cronbach’s alpha coefficient for the MSQ in this study was 0.973, indicating high internal consistency reliability. Additionally, the Kaiser–Meyer–Olkin (KMO) measure of sampling adequacy yielded a value of 0.976, suggesting good construct validity and suitability of the scale.

### 2.5 Turnover intention

Respondents’ intention to leave was measured using the Chinese version of the Intention to Leave Questionnaire developed by Mobley (37). This instrument has been validated and applied in studies among Chinese healthcare workers (38). The scale includes four items: “thought about leaving the current organization,” “thought about leaving the industry,” “actively looking for a new job recently,” and “planning to look for a new job next year.” Responses are rated on a 5-point Likert scale, and scores are summed, with higher totals indicating stronger turnover intention. The scale showed high reliability in this study, with a Cronbach’s *α* of 0.915, indicating high internal consistency reliability. Additionally, the Kaiser–Meyer–Olkin (KMO) value of 0.726 demonstrated excellent sampling adequacy for the scale used, affirming its strong construct validity.

### 2.6 Statistical analyses

SPSS 25.0 was used for statistical analysis. Normality tests were conducted on quantitative data, which were described using mean ± standard deviation when normally distributed. Independent samples *t*-tests and one-way ANOVA were performed to compare group means. Multiple linear stepwise regression analyzed the effects of demographic characteristics, work factors, burnout, and job satisfaction on turnover intention. Spearman correlation assessed bivariate relationships among the main variables.

To minimize sociodemographic variables—including age, gender, education, professional title, employment status, tenure, and type of institution—were included as covariates in the regression and mediation analyses. Variance inflation factors (VIF) were calculated to assess multicollinearity and potential common method bias.

A mediation analysis was conducted using AMOS 28.0 in which turnover intention served as the dependent variable, burnout as the independent variable, and job satisfaction acted as the mediator. A path diagram and the corresponding equations are presented in Figure 1. Bootstrapping with 5,000 resamples in SPSS tested the mediation effect, with significance determined by whether the 95% confidence interval excluded zero. The significance level was set at *α* = 0.05 to ensure statistical rigor.

## 3 Results

### 3.1 Baseline characteristics of the participants

The demographic and sociological characteristics of the sample are summarized in Table 1. Female (66.59%) respondents significantly outnumbered males (33.41%). The age distribution showed concentration in the middle age groups. Most participants held associate or bachelor’s degrees, while those with a master’s or doctorate were relatively few. The distribution of technical titles followed a typical pyramid structure, with junior and untitled staff making up 58.14%, and only 5.86% holding vice-senior or senior titles. Monthly income was mostly concentrated at 3,000 yuan or below, followed by the 3,001–5,000 yuan range. The majority had more than 10 years of work experience. The ratio of formal to non-formal employment was nearly balanced, and 12.31% of respondents reported having an administrative role.

**Table 1:** Comparison of turnover intention scores across sociodemographic groups.

Variables		*N*	%	Turnover intention		
				*M ± SD*	*t/F*	*p*
Gender	Male	2,247	33.41	1.717 ± 0.841	−3.583	0.000***
	Female	4,478	66.59	1.642 ± 0.791		
Age group	≤25	651	9.68	1.675 ± 0.819	0.971	0.457
	26 ~ 30	869	12.92	1.710 ± 0.808		
	31 ~ 35	1,092	16.24	1.699 ± 0.818		
	36 ~ 40	913	13.58	1.643 ± 0.816		
	41 ~ 45	1,190	17.70	1.635 ± 0.802		
	46 ~ 50	1,133	16.85	1.652 ± 0.794		
	51 ~ 55	671	9.98	1.659 ± 0.811		
	56 ~ 60	158	2.35	1.708 ± 0.800		
	≥61	48	0.71	1.629 ± 0.834		
Education	Tech Sch	1882	27.99	1.661 ± 0.816	3.117	0.056*
	Associate	2,814	41.84	1.666 ± 0.817		
	Bachelor	2021	30.05	1.671 ± 0.789		
	Master	4	0.06	2.150 ± 0.943		
	Doctor	4	0.06	2.300 ± 0.346		
Technical title	Senior	35	0.52	1.520 ± 0.763	5.054	0.000***
	Vice-senior	359	5.34	1.589 ± 0.751		
	Intermediate	1,050	15.61	1.620 ± 0.768		
	Junior	1992	29.62	1.737 ± 0.819		
	Primary	1,371	20.39	1.657 ± 0.817		
	No title	1918	28.52	1.644 ± 0.820		
Administrative role (yes/no)	Yes	828	12.31	1.622 ± 0.835	1.722	0.085*
	No	5,897	87.69	1.673 ± 0.805		
Monthly income (¥^a^)	≤3,000	3,525	52.42	1.739 ± 0.829	19.925	0.000***
	3,001 ~ 5,000	2,149	31.96	1.583 ± 0.788		
	5,001 ~ 8,000	899	13.37	1.604 ± 0.759		
	≥8,001	152	2.26	1.561 ± 0.760		
Employment status (yes/no)	Yes	3,260	48.48	1.617 ± 0.788	−4.944	0.000***
	No	3,465	51.52	1.714 ± 0.824		
Years of work	≤2	723	10.75	1.584 ± 0.781	5.135	0.002***
	3–5	900	13.38	1.737 ± 0.829		
	6–9	874	13.00	1.689 ± 0.826		
	≥10	4,228	62.87	1.662 ± 0.804		
Night shift (yes/no)	Yes	2,252	33.49	1.714 ± 0.830	−3.339	0.001***
	No	4,473	66.51	1.643 ± 0.797		

^a^1¥ = US $0.14; Tech Sch, technical school; *p < 0.10; **p < 0.05; ***p < 0.01.

### 3.2 Group differences by sociodemographic characteristics

#### 3.2.1 Turnover intention differences by sociodemographic characteristics

Turnover intention varied significantly across groups (Table 1). Male workers reported higher mean turnover intention scores (1.717 ± 0.841) than females (1.642 ± 0.791, *p* < 0.001). Non-formally employed staff also showed higher values (1.714 ± 0.824) than formal employees (1.617 ± 0.788, *p* < 0.001). Income differences were pronounced: those earning ≤3,000 yuan reported the highest intention (1.739 ± 0.829), whereas the 3,001–5,000 yuan group showed lower scores (1.583 ± 0.788, *p* < 0.01). Workers with higher technical titles reported less intention to leave, with the lowest among senior staff (1.520 ± 0.763). Night-shift workers scored higher (1.714 ± 0.830) than those without night duties (1.643 ± 0.797, *p* = 0.001).

#### 3.2.2 Burnout differences by sociodemographic characteristics

Table 2 shows that males scored significantly higher than females on Emotional Exhaustion (EE = 2.485 ± 1.058) and Depersonalization (DP = 2.164 ± 1.067) than females (EE = 2.353 ± 0.936; DP = 2.060 ± 0.944, *p* < 0.001), while females had higher Perceived Achievement (PA) scores (4.368 ± 1.450). EE increased with age, and PA was higher among younger groups. Master’s degree holders reported the highest EE scores (3.143 ± 1.251), while doctoral holders had the highest DP (2.607 ± 1.192); PA was highest in the master’s group (4.750 ± 1.377) and lowest in the doctoral group (3.893 ± 2.011). Senior title holders scored highest across all three dimensions (EE = 2.657 ± 1.038; DP = 2.268 ± 1.050; PA = 4.550 ± 1.530, *p* < 0.001), reflecting greater burnout. Respondents earning 5,001–8,000 yuan showed more EE, while those earning ≤3,000 yuan had stronger PA. EE was highest in those with over 10 years of service (2.464 ± 0.967), and DP peaked in the 3–5 years group (2.213 ± 1.091). Night shift workers scored significantly higher on EE (2.588 ± 1.031) and DP (2.180 ± 1.036) but lower on PA (4.173 ± 1.473, *p* = 0.001) compared with those without night shifts (EE = 2.301 ± 0.94; DP = 2.052 ± 0.960; PA = 4.420 ± 1.473). Formally employed staff had lower DP (2.060 ± 0.957) and higher PA (4.223 ± 1.432) than non-formal staff (DP = 2.127 ± 1.015; PA = 4.444 ± 1.511).

**Table 2:** Comparison of burnout scores across sociodemographic groups.

Variables		Emotional exhaustion			Depersonalization			Perceived achievement		
		*M* ± *SD*	*t/F*	*p*	*M* ± *SD*	*t/F*	*p*	*M* ± *SD*	*t/F*	*p*
Gender	Male	2.485 ± 1.058	−5.228	0.000	2.164 ± 1.067	−4.087	0.000	4.276 ± 1.529	2.398	0.017
	Female	2.353 ± 0.936			2.060 ± 0.944			4.368 ± 1.450		
Age group	≤25	2.260 ± 1.042	5.815	0.000	2.232 ± 1.113	6.947	0.000	4.551 ± 1.604	7.86	0.000
	26 ~ 30	2.291 ± 0.988			2.173 ± 1.053			4.471 ± 1.594		
	31 ~ 35	2.383 ± 1.002			2.210 ± 1.066			4.466 ± 1.452		
	36 ~ 40	2.366 ± 0.928			2.058 ± 0.981			4.408 ± 1.492		
	41 ~ 45	2.423 ± 0.941			2.003 ± 0.909			4.177 ± 1.435		
	46 ~ 50	2.490 ± 0.973			2.032 ± 0.899			4.195 ± 1.356		
	51 ~ 55	2.523 ± 0.985			2.005 ± 0.889			4.198 ± 1.413		
	56 ~ 60	2.420 ± 0.989			2.048 ± 0.963			4.243 ± 1.575		
	≥61	2.360 ± 1.073			2.006 ± 1.023			4.289 ± 1.235		
Education	Tech Sch	2.352 ± 0.989	8.156	0.000	2.039 ± 0.972	5.039	0.000	4.367 ± 1.515	4.652	0.001
	Associate	2.354 ± 0.982			2.084 ± 0.988			4.399 ± 1.484		
	Bachelor	2.495 ± 0.963			2.158 ± 0.997			4.223 ± 1.425		
	Master	3.143 ± 1.251			3.143 ± 1.017			4.75 ± 1.377		
	Doctor	2.857 ± 0.962			2.607 ± 1.192			3.893 ± 2.011		
Technical title	Senior	2.657 ± 1.038	22.050	0.000	2.220 ± 1.072	3.059	0.010	4.551 ± 1.525	12.608	0.000
	Vice-senior	2.596 ± 0.936			2.002 ± 0.844			3.973 ± 1.293		
	Intermediate	2.529 ± 0.940			2.128 ± 0.957			4.231 ± 1.362		
	Junior	2.466 ± 0.970			2.136 ± 0.979			4.260 ± 1.396		
	Primary	2.382 ± 0.997			2.107 ± 1.008			4.388 ± 1.539		
	No title	2.221 ± 0.982			2.039 ± 1.019			4.504 ± 1.582		
Administrative role (yes/no)	Yes	2.384 ± 1.000	0.413	0.679	2.073 ± 0.997	0.664	0.507	4.195 ± 1.522	2.950	0.003
	No	2.399 ± 0.978			2.097 ± 0.986			4.357 ± 1.470		
Monthly income (¥^a^)	≤3,000	2.381 ± 0.998	7.716	0.000	2.119 ± 1.030	2.259	0.080	4.453 ± 1.501	19.613	0.000
	3,001 ~ 5,000	2.361 ± 0.963			2.053 ± 0.911			4.267 ± 1.461		
	5,001 ~ 8,000	2.541 ± 0.963			2.106 ± 1.007			4.079 ± 1.396		
	≥8,001	2.402 ± 0.836			2.046 ± 0.908			4.164 ± 1.365		
Employment status (yes/no)	Yes	2.412 ± 0.962	1.258	0.209	2.060 ± 0.957	−2.770	0.006	4.223 ± 1.432	−6.149	0.000
	No	2.382 ± 0.997			2.127 ± 1.015			4.444 ± 1.511		
Years of work	≤2	2.151 ± 1.001	24.361	0.000	2.107 ± 1.060	5.913	0.001	4.417 ± 1.629	9.343	0.000
	3–5	2.322 ± 0.978			2.213 ± 1.091			4.525 ± 1.535		
	6–9	2.353 ± 0.992			2.134 ± 1.007			4.419 ± 1.510		
	≥10	2.464 ± 0.967			2.059 ± 0.945			4.266 ± 1.425		
Night shift (yes/no)	Yes	2.588 ± 1.031	−11.095	0.000	2.180 ± 1.036	−4.896	0.000	4.173 ± 1.473	6.495	0.000
	No	2.301 ± 0.940			2.052 ± 0.960			4.420 ± 1.473		

^a^1¥ = US $0.14; Tech Sch, technical school.

#### 3.2.3 Job satisfaction differences by sociodemographic characteristics

Significant differences in job satisfaction were observed across subgroups of primary public health workers (Table 3). Females reported higher intrinsic (4.922 ± 0.832) and extrinsic (11.823 ± 2.509) satisfaction than males (4.878 ± 0.893; 11.612 ± 2.789, *p* = 0.002), with a greater gap in external satisfaction (*p* = 0.002). Satisfaction declined with age, with the ≤25 group scoring highest intrinsic (5.028 ± 0.875) and extrinsic (12.435 ± 2.688) satisfaction (*p* < 0.001). Education showed a nonlinear trend, with internal satisfaction lowest among master’s degree holders (4.15 ± 0.681). Differences by technical title were significant (*p* < 0.001); senior staff had the highest internal satisfaction (5.034 ± 0.896), while those with no title reported the highest external satisfaction (12.242 ± 2.697). Those earning ≥8,001 yuan exhibited the highest satisfaction (intrinsic = 5.130 ± 0.724; extrinsic = 12.528 ± 2.216), whereas those earning ≤3,000 yuan scored lowest (4.828 ± 0.891; 11.54 ± 2.664, *p* < 0.001). Administrative staff showed higher internal but lower external satisfaction (*p* < 0.001). Greater satisfaction was also found among those with higher income, ≤2 years of work, and no night shift duties.

**Table 3:** Comparison of job satisfaction scores across sociodemographic groups.

Variables		Intrinsic job satisfaction			Extrinsic job satisfaction		
		*M* ± *SD*	*t/F*	*p*	*M* ± *SD*	*t/F*	*p*
Gender	Male	4.878 ± 0.893	2.026	0.043**	11.612 ± 2.789	3.134	0.002***
	Female	4.922 ± 0.832			11.823 ± 2.509		
Age group	≤25	5.028 ± 0.875	3.181	0.001***	12.435 ± 2.688	16.418	0.000***
	26 ~ 30	4.941 ± 0.892			12.177 ± 2.647		
	31 ~ 35	4.927 ± 0.859			11.923 ± 2.656		
	36 ~ 40	4.914 ± 0.857			11.814 ± 2.607		
	41 ~ 45	4.879 ± 0.855			11.547 ± 2.544		
	46 ~ 50	4.851 ± 0.820			11.323 ± 2.512		
	51 ~ 55	4.885 ± 0.800			11.350 ± 2.493		
	56 ~ 60	4.815 ± 0.882			11.486 ± 2.442		
	≥61	4.742 ± 0.781			11.529 ± 2.173		
Education	Tech Sch	4.860 ± 0.904	3.665	0.036**	11.598 ± 2.684	4.557	0.018**
	Associate	4.929 ± 0.845			11.869 ± 2.610		
	Bachelor	4.924 ± 0.813			11.743 ± 2.520		
	Master	4.150 ± 0.681			8.150 ± 2.397		
	Doctor	4.300 ± 0.577			11.25 ± 1.636		
Technical title	Senior	5.034 ± 0.896	11.912	0.000***	12.143 ± 2.475	20.475	0.000***
	Vice-senior	4.982 ± 0.731			11.531 ± 2.461		
	Intermediate	4.885 ± 0.814			11.552 ± 2.444		
	Junior	4.826 ± 0.826			11.443 ± 2.554		
	Primary	4.861 ± 0.882			11.719 ± 2.623		
	No title	5.021 ± 0.888			12.242 ± 2.697		
Administrative role (yes/no)	Yes	5.021 ± 0.864	4.102	0.000***	12.255 ± 2.652	−5.939	0.000***
	No	4.891 ± 0.850			11.682 ± 2.593		
Monthly income (¥^a^)	≤3,000	4.828 ± 0.891	25.743	0.000***	11.540 ± 2.664	20.393	0.000***
	3,001 ~ 5,000	4.970 ± 0.813			11.953 ± 2.545		
	5,001 ~ 8,000	5.032 ± 0.779			11.977 ± 2.512		
	≥8,001	5.130 ± 0.724			12.528 ± 2.216		
Employment status (yes/no)	Yes	4.925 ± 0.830	1.699	0.089*	11.74 ± 2.567	−0.376	0.707
	No	4.890 ± 0.874			11.764 ± 2.645		
Years of work	≤2	5.090 ± 0.895	13.053	0.000***	12.712 ± 2.661	53.718	0.000***
	3–5	4.930 ± 0.885			12.110 ± 2.647		
	6–9	4.911 ± 0.888			11.899 ± 2.719		
	≥10	4.870 ± 0.827			11.482 ± 2.515		
Night shift (yes/no)	Yes	4.850 ± 0.842	3.911	0.000***	11.397 ± 2.638	7.973	0.000***
	No	4.936 ± 0.857			11.932 ± 2.574		

^a^1¥ = US $0.14; Tech Sch, technical school; *p < 0.10; **p < 0.05; ***p < 0.01.

### 3.3 Regression analysis of factors influencing turnover intention

This study employed multiple linear stepwise regression with the total turnover intention score as the dependent variable. Independent variables included age, gender, education, years of work, administrative role, as well as indicators of burnout and job satisfaction. The analysis was designed to identify potential predictors of turnover intention and to generate empirical evidence for targeted workforce interventions. Variable coding is detailed in Table 4.

**Table 4:** Variable coding for the multiple linear stepwise regression analysis.

Variables	Coding/operational definition
Gender	Male = 1; Female = 2
Age group	≤25 = 1; 26 ~ 30 = 2; 31 ~ 35 = 3; 36 ~ 40 = 4; 41 ~ 45 = 5; 46 ~ 50 = 6; 51 ~ 55 = 7; 56 ~ 60 = 8; ≥61 = 9
Education	Technical School = 1; Associate = 2; Bachelor = 3; Master = 4; Doctor = 5
Technical title	Senior = 1; Vice-Senior = 2; Intermediate = 3; Junior = 4; Primary = 5; No title = 6
Administrative role (yes/no)	Yes = 1; No = 2
Monthly income (¥^a^)	≤3,000 = 1; 3,001 ~ 5,000 = 2; 5,001 ~ 8,000 = 3; ≥8,001 = 4
Employment status (yes/no)	Yes = 1; No = 2
Years of work	≤2 = 1; 3–5 = 2; 6–9 = 3; ≥10 = 4
Night shift (yes/no)	Yes = 1; No = 2
Burnout	Continuous variable
Job satisfaction	Continuous variable
Turnover intention	Continuous variable

^a^1¥ = US $0.14.

Table 5 shows that stepwise regression identified six significant predictors from 11 variables: burnout, job satisfaction, monthly income, employment status, gender, and age, excluding education, technical title, job type, years of work, and night shift. Burnout and satisfaction had strong explanatory power; income, employment status, and gender reflected social structure; age likely reflected life stage or experience differences.

**Table 5:** Summary of multiple linear stepwise regression results.

Method	Stepwise selection
All variable situation	Job satisfaction, burnout, gender, age, education, technical titles, monthly income (¥^a^), administration role (yes/no), employment status (yes/no), years of work, night shift (yes/no)
Retained variables	Burnout, job satisfaction, monthly income (¥^a^), employment status (yes/no), gender, age
Excluded variables	Education, technical titles, administration role (yes/no), years of work, night shift (yes/no)

Table 6 presents the results of the stepwise regression model. Variance inflation factor (VIF) diagnostics confirmed no multicollinearity among the predictors (all VIF < 3). All predictors in the stepwise regression model had statistically significant effects on total turnover intention (*p* < 0.05). Burnout had the strongest positive association (*β* = 0.337, *p* < 0.001), while job satisfaction showed a significant negative effect (*β* = −0.239, *p* < 0.001).

**Table 6:** Multiple linear stepwise regression analysis of factors associated with turnover intention (*n* = 6,725).

Variables	Unstandardized coefficients		Standardized coefficients	*t*	*p*	*VIF*	*R* ^2^	Adjusted *R*^2^	*F*
	*B*	*SE*	*β*						
Constant	1.834	0.098	0	18.663	0.000***	-	0.269	0.269	*F* = 412.713 *p* = 0.000***
Burnout	0.069	0.003	0.337	26.571	0.000***	1.483			
Job satisfaction	−0.058	0.003	−0.239	−18.654	0.000***	1.515			
Monthly income(¥^a^)	−0.036	0.012	−0.035	−3.063	0.002***	1.195			
Employment status (yes/no)	0.046	0.019	0.029	2.426	0.015**	1.279			
Gender	−0.054	0.018	−0.031	−2.899	0.004***	1.070			
Age	−0.012	0.005	−0.028	−2.491	0.013**	1.199			

^a^1¥ = US $0.14; *p < 0.10; **p < 0.05; ***p < 0.01.

### 3.4 Descriptive statistics and correlation analysis of various variables

Pearson correlation analysis was used to examine the pairwise associations among burnout, job satisfaction, and turnover intention. Burnout was significantly negatively correlated with job satisfaction (*r* = −0.557, *p* < 0.001) and positively correlated with turnover intention (*r* = 0.475, *p* < 0.001). Job satisfaction was also negatively correlated with turnover intention (*r* = −0.429, *p* < 0.001). Results are shown in Table 7.

**Table 7:** The correlations among burnout, job satisfaction and turnover intention.

Variables	Burnout	Job satisfaction	Turnover intention
Burnout	1		
Job satisfaction	−0.557***	1	
Turnover intention	0.475***	−0.429***	1

*p < 0.10; **p < 0.05; ***p < 0.01.

### 3.5 The mediating role of job satisfaction between burnout and turnover intention

**Figure 2:**
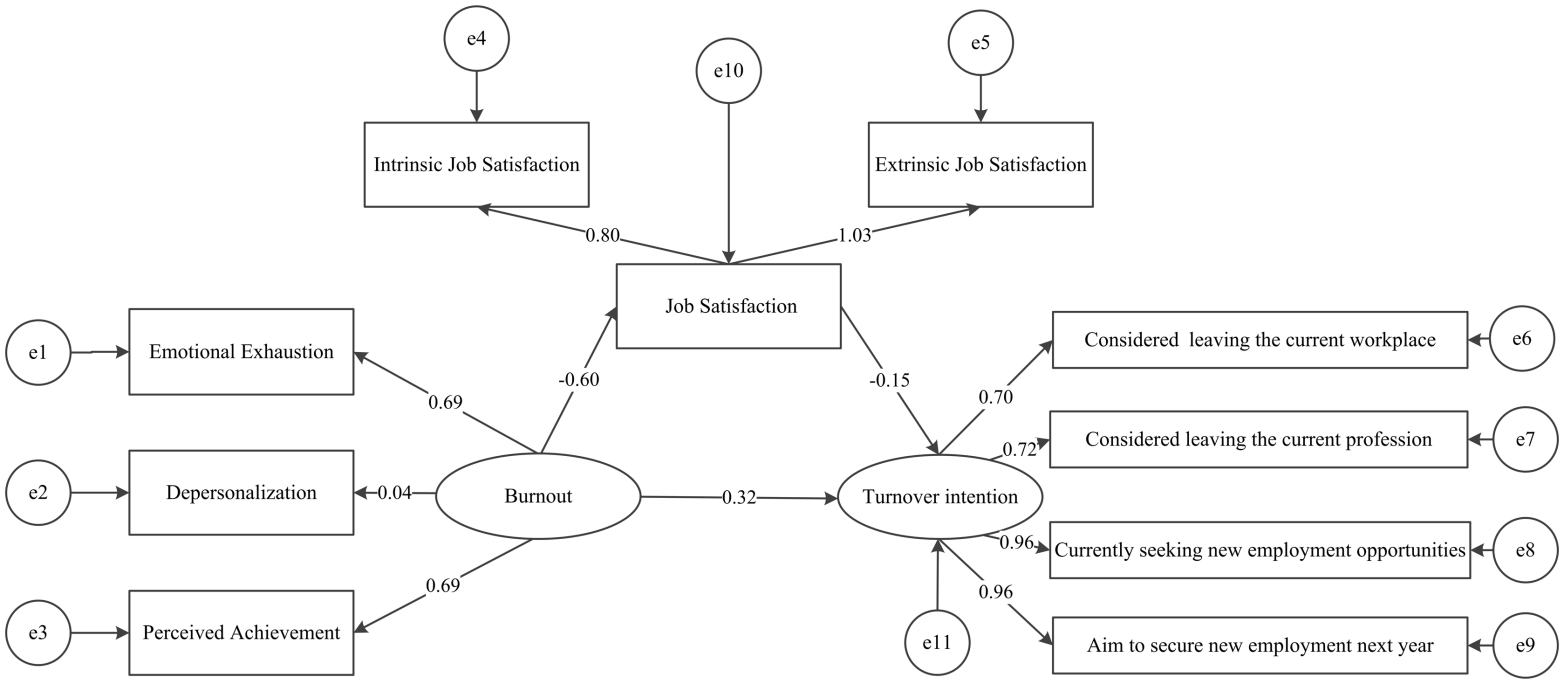
The mediating model of burnout, job satisfaction, and turnover intention.

This study employed a structural equation model to test the mediating path of “burnout → job satisfaction → turnover intention,” with burnout as the independent variable, job satisfaction as the mediator, and turnover intention as the dependent variable (Figure 2).

All path coefficients were statistically significant (*p* < 0.001), as shown in Table 8. Specifically, burnout significantly negatively predicted job satisfaction (*β* = −0.602, *SE* = 0.15, *CR* = −29.739); job satisfaction negatively predicted turnover intention (*β* = −0.148, *SE* = 0.2, *CR* = −9.108); and burnout had a significant direct positive effect on turnover intention (*β* = 0.32, *SE* = 0.2, *CR* = 14.747).

**Table 8:** The path coefficients of the mediation model.

Paths	*B*	*β*	*SE*	*CR*	*p*
Burnout → job satisfaction	−0.456	−0.602	0.15	−29.739	<0.001
Job satisfaction → turnover intention	−0.183	−0.148	0.2	−9.108	<0.001
Burnout → turnover intention	0.298	0.32	0.2	14.747	<0.001

In addition, the 95% confidence interval generated using the Bootstrap method did not contain zero, indicating a significant mediation effect (Table 9). The results show that burnout affects turnover intention through both direct and indirect paths, reflecting a dual mechanism. The indirect effect was significant (effect = 0.089, *SE* = 0.012), suggesting that job satisfaction partially mediates the relationship between burnout and turnover intention. The direct effect was also significant (effect = 0.32, *SE* = 0.027), accounting for 78% of the total effect. The total effect was significant (effect = 0.409, *SE* = 0.021), with 21.76% contributed by the indirect path and 78.24% by the direct path, highlighting the multi-path nature of burnout’s impact on turnover intention.

**Table 9:** Decomposition of direct, indirect, and total effects of job satisfaction in the mediation model.

Measure	Effect size	*SE*	Bootstrap 95% CI			Effect proportion
			Lower	Upper	*p*	
Indirect effect	0.089	0.012	0.064	0.115	0.001	22%
Direct effect	0.32	0.027	0.265	0.374	0.001	78%
Total effect	0.409	0.021	0.366	0.449	0.001	100%

## 4 Discussion

The study revealed that burnout was positively associated with turnover intention and served as the strongest positive predictor. Burnout was also negatively associated with job satisfaction, which, in turn, showed a significant negative relationship with turnover intention. Furthermore, job satisfaction partially mediated the relationship between burnout and turnover intention. These results indicate that burnout directly increases turnover intention and indirectly contributes to it by eroding job satisfaction and professional identity through heightened emotional exhaustion and anxiety.

### 4.1 Discussion of results

First, correlation analysis and structural equation modeling confirmed a significant positive association between burnout and turnover intention, thereby supporting the first hypothesis. This finding is consistent with previous research (15, 39, 40). As the backbone of routine health management and emergency response, primary public health workers are central to the “Healthy China” strategy. However, low pay, limited promotion opportunities, and excessive workloads remain major drivers of turnover intention (41). The present study further found that factors such as technical title, income, employment status, tenure, and night shifts significantly influenced turnover (42). From an SDT perspective, unmet psychological needs for autonomy, competence, and relatedness contribute to burnout among primary public health workers. In the current healthcare context, weak patient–provider trust and limited professional recognition undermine these needs, leading to emotional exhaustion, lower job satisfaction, and increased turnover risk.

Second, job satisfaction was negatively associated with turnover intention, supporting the second hypothesis. Similar findings have been reported among nurses in Australia and primary care workers in the UK (43, 44). Job satisfaction is not only essential to the career development of primary public health workers but also a cornerstone for an efficient public health system. However, heavy workloads, constrained resources, and promotion barriers contribute to persistently low satisfaction levels (45). Male workers tended to report lower satisfaction and higher turnover intention, possibly reflecting greater career ambition and heightened frustration with stagnant advancement prospects. From the perspective of SET, job satisfaction reflects the perceived balance between effort (e.g., workload, skills) and rewards (e.g., pay, recognition, and promotion). When perceived returns fail to meet expectations, satisfaction declines and turnover risk rises. Workers with longer tenure, lower professional titles, and fewer advancement opportunities are especially vulnerable, as they often face constrained career pathways, limited access to training, and a lack of long-term professional development planning (28, 46). This problem is especially severe in rural township hospitals, where structural and resource constraints are most pronounced (47, 48). Psychologically, reduced job satisfaction weakens emotional commitment and undermines alignment with organizational goals, leading to motivational fatigue and heightened uncertainty about the future (49). Such dynamics not only intensify individual turnover intention but also exacerbate systemic instability, forming a vicious cycle of attrition. Enhancing job satisfaction and professional identity is thus essential to sustaining service motivation and public health system resilience.

Finally, job satisfaction was found to play a significant partial mediating role between burnout and turnover intention among primary public health workers, confirming the third hypothesis. Approximately 21.76% of the total effect of burnout on turnover intention was transmitted through job satisfaction, consistent with previous findings that highlight its central role in the burnout–turnover mechanism. However, other unobserved psychological or contextual factors may also contribute to this process. Similar evidence from rural China indicates that professional identity and job satisfaction jointly mediate the impact of burnout on turnover intention (50). Specifically, negative experiences such as emotional exhaustion may erode professional identity and service motivation, thereby lowering job satisfaction and increasing turnover risk—ultimately undermining the efficiency and quality of primary public health systems. In the first stage of the mediation pathway, burnout was significantly negatively correlated with job satisfaction (*r* = −0.557, *p* < 0.001), indicating that heightened burnout leads to lower satisfaction. This is consistent with previous research (16, 51). In the second half, job satisfaction was also negatively associated with turnover intention (*r* = −0.429, *p* < 0.001), reaffirming its role as a key antecedent of turnover, as reported in studies of healthcare workers in both China and Europe (52, 53). The JD-R model helps explain these findings: high job demands increase burnout by draining energy, while limited resources reduce motivation and satisfaction. Burnout-related turnover is therefore not driven by dissatisfaction alone but also by structural pressures in the primary health system. Public health workers are often marginalized as “second-tier doctors,” with undervalued contributions and restricted career growth. Weak systems for education, promotion, and recognition further erode their sense of belonging and confidence. Improving pay, promotion pathways, and social recognition is essential to reduce burnout and enhance workforce stability.

### 4.2 Theoretical and practical implications

This study has theoretical and practical implications. Theoretically, research on burnout, job satisfaction, and turnover intention has largely focused on corporate, academic, and tertiary hospital staff, with limited evidence from primary public health workers (11, 31, 54, 55). Grounded in the JD-R model, together with SET and SDT (22–24), this study proposes an integrated framework linking burnout, job satisfaction and turnover intention. It highlights how high job demands and scarce resources jointly shape these relationships. The framework clarifies the psychological pathways from burnout to turnover and extends existing theories to China’s primary public health context, which is marked by chronic resource shortages and multiple role demands.

Practically, our findings highlight the need for tailored interventions to stabilize the primary public health workforce and address differences in burnout, satisfaction, and turnover. We propose a three-level strategy. At the individual level, priority should be given to psychological support, career development, and strengthening professional identity. Targeted mental health services and optimized shift schedules are recommended for high-risk groups such as younger and night-shift staff. Flexible policies, childcare, and research support can help improve work–life balance, particularly for highly educated women. At the organizational level, reforming compensation and promotion systems can enhance fairness and motivation. Better communication, mental health resources, and greater clinical autonomy may also reinforce professional identity. At the societal level, improving public recognition, working conditions, and incentive systems can help reduce attrition. Together, these multi-level approaches can alleviate burnout, decrease turnover, and enhance the sustainability of primary public health systems.

### 4.3 Shortcomings and prospects

This study has several limitations. First, the cross-sectional design limits the ability to infer causality between burnout, job satisfaction, and turnover intention. Although our analysis identifies plausible psychological pathways, the temporal and causal directions cannot be definitively established. Future studies should employ longitudinal, multilevel, or quasi-experimental designs to verify the temporal sequence and causal mechanisms identified in this research. Second, the sample was drawn from four cities within the Huaihai Economic Zone, which may limit the generalizability of the findings. Future research should employ broader cross-regional and cross-country, multi-stage stratified sampling to examine the robustness of these results across areas with diverse socioeconomic conditions, health resource availability, and cultural contexts. Finally, regional and national differences may further constrain generalizability, and the applicability of these findings to other settings requires additional empirical validation.

## 5 Conclusion

This study, grounded in the Job Demands–Resources (JD-R) model, examined the relationships among turnover intention, burnout, and job satisfaction among primary public health workers. Burnout emerged as the strongest predictor of turnover intention, with job satisfaction partially mediating this relationship. Gender, age, and income also significantly influenced all three outcomes. These findings highlight the need to strengthen resource provision, enhance job satisfaction, and implement multi-level interventions to reduce burnout and stabilize the primary public health workforce.

## Data Availability

The raw data supporting the conclusions of this article will be made available by the authors, without undue reservation.
